# Psychometric testing of the short version of the world health organization quality of life (WHOQOL-BREF) questionnaire among pulmonary tuberculosis patients in Taiwan

**DOI:** 10.1186/1471-2458-12-630

**Published:** 2012-08-09

**Authors:** Wei-Sheng Chung, Yu-Ling Lan, Ming-Chin Yang

**Affiliations:** 1 Department of Internal Medicine, Taichung Hospital, Department of Health, the Executive Yuan, Taichung, Taiwan; 2 Institute of Health Industry Management, Central Taiwan University of Science and Technology, Taichung, Taiwan; 3 Department of Counseling and Clinical Psychology, National Dong Hwa University, Hualien, Taiwan; 4 Institute of Health Policy and Management, College of Public Health National Taiwan University, Taipei, Taiwan

**Keywords:** Quality of life, Patients with tuberculosis, Instrumentation, Validity, Reliability

## Abstract

**Background:**

Studies on the effects of tuberculosis on a patient’s quality of life (QOL) are scant. The objective of this study was to evaluate the psychometric properties of the Taiwan short version of the World Health Organization Quality of Life (WHOQOL-BREF) questionnaire using patients with tuberculosis in Taiwan and healthy referents.

**Methods:**

The Taiwanese short version of the WHOQOL-BREF was administered to patients with tuberculosis undergoing treatment and healthy referents from March 2007 to July 2007. Patients with tuberculosis (*n* = 140) and healthy referents (*n* = 130), matched by age, sex, and ethnicity, agreed to an interview. All participants lived in eastern Taiwan. Reliability assessments included internal consistency, whereas validity assessments included construct validity, convergent validity, and discriminant validity.

**Results:**

More than half of these patients and referents were men (70.7% and 66.2%, respectively), and their average ages were 50.1 and 47.9 years, respectively. Approximately 60% of patients and referents were aboriginal Taiwanese (60.7% and 61.1%, respectively). The proportion with low socioeconomic status was greater for these patients. The internal consistency reliability coefficients were .92 and .93 for the patients and healthy referents, respectively. Exploratory factor analysis on the healthy referents displayed a 4-domain model, which was compatible with the original WHOQOL-BREF 4-domain model. However, for the TB patient group, after deleting 3 items, both exploratory and confirmatory factor analysis revealed a 6-domain model.

**Conclusion:**

Psychometric evaluation of the Taiwan short version of the WHOQOL-BREF indicates that it has adequate reliability for use in research with TB patients in Taiwan. However, the factor structure generated from this TB patient sample differed from the WHO’s original 4-factor model, which raised a validity concern to apply the Taiwan short version of the WHOQOL-BREF to Taiwanese TB patients. Future research recruiting another sample to revisit this validity issue must be conducted to determine the validity of the WHOQOL-BREF TW in patients with TB.

## Background

Tuberculosis (TB) remains a serious public health, social, and economic problem worldwide. Several studies have concentrated on general clinical outcomes [1-4]. TB can result in anatomic destruction and functional change of the lung, despite effective treatment and available cures. A focus group interview with 39 patients with active TB in Canada revealed that TB may affect quality of life in 4 aspects, namely, diagnosis with emotional effect, medication with possible adverse effects, social support and functioning change, and health behavior for people [5]. In Taiwan, patients with TB should be mandatorily notified to TB registry in Taiwan Centers of Disease (CDC). Infectious TB patients may be hospitalized or isolated from the public. TB results in not only disease-related health problems, but also psychological dissatisfaction and social stigma [6].

However, the effect on patient quality of life (QOL) is rarely quantified. Specifically, the effect of TB on a patient’s global QOL (physical, mental, or social impairment) has not been studied adequately, particularly in developed countries [7-11]. Evaluation of the TB disease effect on a patient’s QOL requires selecting a proper QOL measure to assess TB patients.

Quality of life is defined as people’s perceptions of their position in life in the context of the culture and value systems in which they live, and in relation to their goals, expectations, standards, and concerns [12]. Numerous instruments are available to assess this concept. The 2 most well known QOL instruments are the Medical Outcome Study SF-36 and the World Health Organization Quality of Life-BREF (WHOQOL-BREF). These 2 instruments have been applied in many countries and different populations, with good reliability and validity [13]. The Taiwan versions of both instruments were found to have acceptable reliability and validity [14,15]. Huang et al. [13] compared the psychometric properties and factor structures of these 2 instruments on a national representative sample (n = 11 440) in Taiwan. They concluded, “The SF-36 and WHOQOL-BREF appear to measure different constructs: the SF-36 measures health-related QOL, while the WHOQOL-BREF measures global QOL” ( [13], p. 15). Therefore, the current study selects the WHOQOL-BREF to examine TB patients’ global QOL. This paper analyzes the psychometric properties of the Taiwan version of the WHOQOL-BREF on TB patients in Taiwan.

The QOL measurement can be based on economic utility (*utility measure*; [16]) or psychometrics (*profile analysis*; [17]). Profile analysis uses a generic or disease-specific questionnaire. In 1991, the WHO initiated a cross-cultural project to develop a standard WHOQOL-100 questionnaire for generic use. The WHOQOL research group later simplified the WHOQOL-100 to a short form, called the WHOQOL-BREF [18,19], which includes 26 items (24 items that represent each of the 24 specific facets of the WHOQOL-100, and 2 global/general items). This instrument can be widely used in interventional studies for cross-cultural, population, or intra-disease comparisons.

The factor structure of the WHOQOL-BREF TW is a 4-factor model that includes physical, psychological, social relationships, and environmental domains to measure a person’s quality of life in these 4 aspects. In Taiwan, this 4-factor model has been validated for the general population and for people with specific diseases, such as those undergoing hemodialysis or those with a traumatic spinal cord injury [20-22]. The Taiwan version (WHOQOL-BREF TW) added 2 national items with permission from the WHOQOL group. These 2 national items were selected from the Taiwan version of the WHOQOL-100 to represent 2 new facets generated from the Taiwan version of the WHOQOL-100 [23].

However, the questionnaire has not been applied to patients with TB, despite TB remaining prevalent in Taiwan. This study examines the psychometric properties of the WHOQOL-BREF TW in patients with TB in Taiwan. In this study, all TB patients were recruited from eastern Taiwan during the study period. The incidence in eastern Taiwan is relatively high: 120.4 per 10^5^ compared with 67.4 per 10^5^ globally in 2006 (Center for Disease Control, 2007). In addition to the endemic TB area, eastern Taiwan is regarded as a disadvantaged region by living standards, medical services, social welfare, and mass transportation [24]. If the psychometric properties of the WHOQOL-BREF TW in TB patients in eastern Taiwan differ significantly from the findings of previous Taiwan studies, possible explanations for these differences might be caused by the TB disease effect or the regional effect.

This study recruits healthy referents in eastern Taiwan to evaluate these 2 effects. First, if the psychometric properties of the WHOQOL-BREF TW among healthy referents from eastern Taiwan were similar to the findings of previous studies among healthy Taiwanese people, the regional effect would be considered insignificant. Consequently, if the psychometric properties of the WHOQOL-BREF TW among TB patients from eastern Taiwan differed from those among healthy referents from eastern Taiwan, the major reason for these differences could be the TB disease effect. The healthy referent group in this study was also used to compare with the TB patient group, to determine if their differences reached a statistically significant level to establish the discriminant validity of the WHOQOL-BREF TW.

More than 50% of the TB patients in this study were aboriginal Taiwanese. Being a minority in Taiwan, aboriginal Taiwanese often face economic disadvantage and discrimination. Chang [25] indicated that many aboriginal Taiwanese have difficulty finding a job. According to the 2003 government statistical reports, the unemployment rate of aboriginal Taiwanese was higher than the national average unemployment rate. Even with a job, approximately half of the aboriginal Taiwanese earned less than US$625 (a basic standard of living) per month [25]. Aboriginal Taiwanese in eastern Taiwan face regional and racial disadvantages. Having TB places aboriginal Taiwanese TB patients in eastern Taiwan under a 3-fold disadvantageous condition -- being a minority, residing at a remote region, and having TB. To ensure that the ethnic effect and regional effect did not affect the psychometric findings of the WHOQOL-BREF TW on TB patients, healthy referents in eastern Taiwan were recruited by matching the ethnic proportion of TB patients in this study.

## Methods

### Participants

A population-based prospective study was conducted in patients diagnosed with TB residing in eastern Taiwan, an area populated by 0.57 million residents. From March 2007 to July 2007, a total of 195 patients were diagnosed and confirmed as having TB by all hospitals located in eastern Taiwan. All patients were reported in the TB registry system and were therefore recruited for the study. Of the patients with TB, 22 had disturbed consciousness, 3 had extrapulmonary tuberculosis, and 6 were repatriates after excluding the diagnosis. Twelve were diagnosed with the disease after death, and 12 refused to be interviewed. The remaining 140 patients with TB (including both inpatients and outpatients) agreed to participate in this study. To compare the psychometric properties of the WHOQOL-BREF TW among patients with TB and findings of the previous study, 130 healthy referents were recruited from the community as a comparison group. These healthy referents were matched by age, sex, and ethnicity, in similar proportions as the patients with TB.

### Procedure

Because this study planned to interview TB patients and healthy referents resided in eastern Taiwan, the Institutional Review Board of Hualien Hospital in eastern Taiwan approved the study, where the first author served during the study period before conducting the study. This study also received an approval to use the TB registry from the Taiwan CDC before administering the WHOQOL-BREF TW to patients with TB undergoing treatment and healthy referents. All participants gave written consent before the interview.

Registered nurses and community health care workers were trained as interviewers and visited participants in the community to complete the WHOQOL-BREF TW. All staff involved in this study signed a letter of agreement to maintain patient confidentiality. Names and addresses of TB patients came from the Taiwan CDC’s TB registry. The 140 patients with TB were interviewed 3 times (initially, at 6 weeks, and at 4 months) to evaluate if their QOL was influenced by the progress of their TB treatment outcomes. TB patients’ initial interviews were conducted within 2 weeks after receiving anti-TB treatment, and the mean time of their initial interview was 8.3 ± 4.8 days after diagnosis. This research also recruited healthy referents from the community screening and matched them by age, sex, and ethnicity, in similar proportions to the TB patients.

### Instruments

The Taiwan version of the WHOQOL-BREF TW consists of 28 items— 26 items of the original WHOQOL-BREF and 2 region-specific/national items. Among the 26 items, 24 domain-specific items represent each of the 24 specific facets of the WHOQOL-100, and are used to measure individual QOL in 4 domains (physical, psychological, social relationships, and environmental domains). The other 2 items are used to measure individual perceptions of global QOL (G1) and health status (G2). The remaining 2 region-specific items (“The feeling of being respected by others” and “Food satisfaction”) were added to the Taiwan version to capture the unique cultural characteristics of Taiwan [23,26]. The WHOQOL-BREF domain scores were calculated by multiplying the average scores of all items in each domain by 4 [18], and ranged from 4 to 20. Higher domain scores indicate a better QOL.

### Data analysis

The following analyses correlated the 2 global QOL items with the 4 QOL domain scores to examine the convergent validity of the WHOQOL-BREF TW, and the remaining 26 items (24 generic items and 2 national items) were used to establish the validity of this instrument. Based on the user manual provided by the WHOQoL-Taiwan Group [23], the reliability of the WHOQOL-BREF TW was examined in three different manners: Version 1 (including 24 generic items only), Version 2 (including 24 generic items and 2 global items), and Version 3 (including 24 generic items, 2 global items, and 2 national items).

Data analyses were performed using SPSS 16.0 and AMOS 6.0. Reliability assessment included internal consistency reliability, which was assessed using Cronbach’s α. Validity assessments included construct validity, convergent validity, and discriminant validity. Construct validity was examined using exploratory factor analysis (EFA), and subsequently analyzed using confirmatory factor analysis (CFA), to determine how well the models fit the data of the TB patients and the healthy referents from eastern Taiwan.

In CFA analyses, the Akaike index (AIC) can be used to compare nested and un-nested models and to rank all the comparative models; the one with the lowest value is then selected as the best. Following the suggestions of Hu and Bentler [27], the relative chi-square (*χ*^2^/df), the comparative fit index (CFI), and the root mean square error of approximation (RMSEA) were used as the model fit indices to evaluate which model best fit the data. Although the chi-square goodness of fit (*χ*^2^) is a widely used fit index, it is sensitive to sample size. If the sample size is large, the chi-square fit index may be significant, even if the difference between the observed and model-implied covariances is small [28]. In contrast to the chi-square fit index, the relative chi-square fit index (*χ*^2^/df) was applied to overcome this problem. If a measurement model fits the data well, the relative chi-square fit index is expected to be less than 3 [29].

The RMSEA is a population-based index, which is relatively independent of sample size, and “assesses the extent to which a model fits reasonably well in the population” ( [30], p. 83). A value < .05 represents good fit, whereas a value < .08 indicates reasonable fit [31]. The CFI “evaluates the fit of a user-specified model in relation to a more restricted, nested baseline model” ( [30], p. 84). A value of .90 or greater is desired [30].

Pearson correlation was used to assess convergent validity and a multivariate analysis of covariance (MANCOVA) for the comparison between TB patients and healthy referents to determine discriminant validity. The use of MANCOVA can help control for Type 1 error rate and avoid having to use a Bonferroni correction in a series of univariate analyses [32].

## Results

Prior to data analyses, data were examined to assess the accuracy of data entry, the percentage of missing values, and the assumption of linearity and normality [32], to ensure the quality of the 2 data sets. Data entry accuracy was examined using the range of data [33]. In this study, 1.7% and 10.3% of data were missing from the healthy referents and TB patients, respectively. Both groups had approximately less than 10% of missing data, which indicated a reasonable missing data rate for the following analyses [33].

Because of missing data on both TB patients and healthy referents, the maximum likelihood estimator was applied to obtain an estimation of CFA measurement models (Brown [30]). The normality assumption was examined through skewness and kurtosis, with absolute values less than one. According to Muthen and Kaplan [34], if variables with skewness and kurtosis are close to −1 and +1, estimating the parameter of non-normal variables by using the maximum likelihood method produces acceptable values. The assumption of linearity among pairs of variables was examined through scatterplot inspection [32]. A nonlinear relationship was not detected in the data from TB patients or healthy referents.

### Demographic characteristics

Of the 140 patients with tuberculosis, the mean age was 50.13 years (*SD* = 18.62); 70.7% of the sample was men, and more than half of them were aboriginal Taiwanese (Table 1). The healthy referents in this study were recruited by matching age, sex, and ethnicity proportions to the TB patient group. The mean age of the 130 healthy referents was 47.91 years (*SD* = 18.94).

**Table 1:** Demographic characteristics of patients with tuberculosis and healthy referents

		**Patients with TB**	**Healthy referents**	***t *or *χ***^***2***^
		**(*n *= 140)**	**(*n *= 130)**	**(*p *value)**
Demographic Variables		*n* (%)	*n* (%)	
Age				
	< 30 year-old	19 (13.6)	27 (21.6)	
	30 to 39 year-old	21 (15.0)	14 (11.2)	
	40 to 49 year-old	31 (22.1)	27 (21.6)	
	50+ year-old	69 (49.3)	57 (45.6)	
	missing	0 (0)	5	
	Mean (SD); Range	50.13 (18.62); 11~89	47.91 (18.94); 11~83	*t* (263) = 0.963
				(*p* = 0.337)
Gender				
	Male	99 (70.7)	86 (66.2)	
	Female	41 (29.3)	44 (33.8)	*χ*^*2*^ (1) = 0.650
				(*p* = 0.434)
Level of Education				
	Illiterate or primary	71 (51.4)	31 (25.4)	
	Junior high	34 (24.6)	20 (16.4)	
	High School	26 (18.8)	37 (30.3)	
	College or graduate	7 (5.1)	34 (27.9)	*χ*^*2*^ (3) = 38.177
				(*p* < 0.001)
	missing	2	8	
Marriage				
	Single	46 (33.1)	30 (23.6)	
	Married	68 (48.9)	74 (58.3)	
	Living as married	12 (8.6)	9 (7.1)	
	Divorced/widowed	13 (9.4)	14 (11.0)	*χ*^*2*^ (3) = 3.553
				(*p* = 0.314)
	missing	1	3	
Ethnicity				
	Min-Nan	38 (27.1)	34 (27.0)	
	Hakka	9 (6.4)	7 (5.6)	
	Mainlander	8 (5.7)	8 (6.3)	
	Aboriginal	85 (60.7)	77 (61.1)	*χ*^*2*^ (3) = 0.131
				(*p* = 0.988)
	missing	0	4	
Personal monthly income				
(in US dollars)				
	< 950	117 (84.8)	86 (66.7)	
	950-1,900	18 (13.0)	28 (21.7)	
	> 1,900	3 (2.2)	15 (11.6)	*χ*^*2*^ (2) = 14.621
				(*p* < 0.001)
	missing	2	1	

*Notes.* TB = tuberculosis.

Chi-squared tests and *t* tests were conducted to examine the differences between the TB patient group and the healthy referent group in major demographic characteristics such as age, sex, and education. As expected, no significant differences emerged between the 2 groups in age (*t* (263) = 0.963, *p* = .337), sex (*χ*^*2*^ (1) = 0.650, *p* = .434), ethnicity (*χ*^*2*^ (3) = 0.131, *p* = .988), and marriage status (*χ*^*2*^ (3) = 3.553, *p* = .314).

Compared with TB patients, more than half of the healthy referents had a high school or college degree, and relatively higher personal incomes than TB patients. Significant differences existed between the 2 groups in level of education (*χ*^*2*^ (3) = 38.177, *p *< .001) and in personal monthly income (*χ*^*2*^ (2) = 14.621, *p *< .001). Descriptive statistics are shown in Table 1.

### Internal consistency reliability

The Cronbach’s alpha values of the WHOQOL-BREF TW total scale and subscales are presented in Table 2. Regardless of the versions of the WHOQOL-BREF TW total scale, the Cronbach’s alpha values of the WHOQOL-BREF TW total scale were all above .91 for TB patients, healthy referents, and all participants. The alpha values of the WHOQOL-BREF TW subscales ranged from .61 to .82 for the TB patient group, from .53 to .87 for the healthy referent group, and from .58 to .85 for all participants. Except for the social relationship subscale, the alpha values of the WHOQOL-BREF TW total and subscales were all larger than 0.7, the lower acceptable bound for an alpha value [35]. These results demonstrate good internal consistency of the WHOQOL-BREF TW among TB patients and healthy referents.

**Table 2:** Cronbach’s coefficient alpha values by the status of participants for the WHOQOL-BREF TW total scale and four subscales

	**TB Patients**	**Healthy Referents**	**All Participants**
	**(N = 140)**	**(N = 130)**	**(N = 270)**
**Subscale**			
Physical	.77	.72	.77
Environmental	.80	.86	.83
Environmental ^1^	.82	.87	.85
Psychological	.81	.71	.76
Social Relationship	.61	.53	.58
Social Relationship^2^	.64	.58	.61
**Total Scale**			
Version 1 (24 items)	.92	.91	.92
Version 2 (26 items)	.92	.92	.92
Version 3 (28 items)	.93	.93	.93

Note. Environmental^1^ subscale includes eight generic items and one national item. Social relationship^2^ subscale includes three generic items and one national item. Total scale (Version 1) only includes 24 generic items. Total scale (Version 2) includes 24 generic items and two global items. Total scale (Version 3) includes 24 generic items, two global items and two national items.

### Construct validity

The Kaiser-Meyer-Olkin (KMO) measure of sampling adequacy and the Bartlett test of sphericity were used prior to factor analysis, to ensure that the data from both patients with TB and healthy referents were appropriate for conducting factor analysis. The KMO measure of sampling adequacy for TB patients and healthy referents was .879 and .887, respectively, indicating that these 2 samples had a sufficient level of factorability. The Bartlett tests of sphericity for both data were significant at the .001 level, indicating that the correlation matrices were not identical to the factor structure matrices. Both tests revealed that data from TB patients and healthy referents were appropriate for factor analysis [32].

When performing factor analysis, the sample size should be at least 250 to 300 cases [32]. However, in the present study, the number of patients with TB in eastern Taiwan was relatively small. Instead of following the general expected sample size, this research adopted the Gorsuch [36] perspective to evaluate the sufficiency of the sample size in this study. The Gorsuch [36] suggestion on a sufficient sample size for factor analysis is that a ratio of 5 participants per item should be present and that the total sample size should include more than 100 participants. Based on the Gorsuch perspective, the sample sizes of TB patients and healthy referents meant that the data were sufficient for factor analysis.

In the EFA of the TB patient data, factor analysis was conducted by principal component analysis, followed by Oblimin rotation with an eigenvalue above 1. Six conceptually meaningful factors were extracted, explaining 66% of the total variance (Table 3). In this 6-factor model, 3 items (Pain, Medical dependency, and Life enjoyment) were excluded because of low factor loadings (lower than 0.3); thus, only 23 items were included. The 6-factor model was a variation on the original WHOQOL-BREF factor structure.

**Table 3:** Factor analysis of WHOQOL-BREF TW data from 140 patients with tuberculosis

**Item**	**Item description**	**F1**	**F2**	**F3**	**F4**	**F5**	**F6**
Factor1 (F1): Self-confirmation (7 items)							
19	Self-satisfaction	.770	.385	.358		.350	.431
7	Concentration	.727			.368	.460	
10	Vitality	.715		.354	.311	.394	
18	Satisfaction with work capacity	.715	.331				.526
20	Satisfaction of personal relationships	.675	.654				.332
11	Acceptance of appearance	.670		.610	.329	.342	
6	Life meaning	.580			.451	.353	.477
Factor2 (F2): Social support (2 items)							
23	Satisfaction with living place		.824	.395			.314
22	Satisfaction with friend support		.800				.415
Factor3 (F3): Psycho-Social-Environmental (5 items)							
9	Physical environmental health		.353	.729		.597	
26	Negative feeling	.560		.681		.350	
27	The feeling of being respected by other		.356	.638			.370
8	Life safety	.559		.638		.492	
28	Food satisfaction		.312	.590			.487
Factor4 (F4): Availability (3 items)							
14	Opportunity for leisure activities	.377	.359		.720	.341	.304
12	Enough money for needs	.333		.355	.619		.537
13	Daily information availability	.489	.460	.547	.618	.326	
Factor5 (F5): Activity (3 items)							
16	Satisfaction with sleep					.864	
17	Satisfaction with ability to perform daily living	.538		.338	.309	.724	.359
15	Ability to get around	.508	.453		.491	.580	.403
Factor6 (F6): Accessibility (3 items)							
25	Satisfaction with transportation	.315	.415				.833
21	Satisfaction with sex life	.306	.323		.343		.730
24	Satisfaction with access to health service		.453			.374	.587
Eigenvalues		5.627	3.72	3.92	2.78	3.725	4.015
Total variance explain (%)		66.044					

*Note:* TB = tuberculosis.

Factor 1 comprised 7 items belonging to 3 original WHOQOL-BREF domains (physical, psychological, and social relationship domains) and was labeled as the *self-confirmation factor* to capture the need for people to confirm the meaning of self. Factors 2 and 6 consisted of items belonging to 2 WHOQOL-BREF original domains (environmental and social relationship domains), and were renamed as the *social support factor* and the *accessibility factor*. Factor 3 included items from 3 WHOQOL-BREF original domains (environmental, psychological, and social relationship domains), and was renamed as the *psycho-social-environmental factor*. Factor 4 (*Availability)* and Factor 5 (*Activity*) included only some items from the original WHOQOL-BREF environmental and physical domains, and can be regarded as the subscales of these 2 original WHOQOL-BREF domains.

For the healthy referents, 5 factors were extracted using principal component analysis with Varimax rotation, accounting for 62.58% of the total variance. Because of parsimony of the factor structure, Factor 5, which included only one item, was excluded from the final model. Therefore, the final EFA factor model for healthy referents consisted of 4 factors that explained 55.69% of the total variance. In this 4-factor model, items 3 (*pain*), 4 (*medical dependency*), 16 (*satisfaction with sleep*), and 22 (*satisfaction with friend support*) were excluded because of low factor loadings (lower than 0.3). This resulted in 22 included items (Table 4). The final EFA model was similar to the WHOQOL-BREF 4-factor model, except for the social relationship domain. The first 3 factors consisted of most items belonging to the corresponding original WHOQOL-BREF domains. The social relationship factor, which included only half of the items belonging to the original WHOQOL-BREF social relationship domain, is a more specific definition of the original WHOQOL-BREF domain.

**Table 4:** Factor analysis of WHOQOL-BREF TW data from 130 healthy referents

**Item**	**Item description**	**F1**	**F2**	**F3**	**F4**	**F5**
Factor1 (F1): Environment (10 items)						
23	Satisfaction with living place	.696				.378
24	Satisfaction with access to health service	.688				.395
25	Satisfaction with transportation	.688				
28	Food satisfaction	.666			.391	
12	Enough money for needs	.641			.330	
13	Daily information availability	.628		.537		
11	Acceptance of appearance	.569	.313		.391	
9	Physical environmental health	.558		.301		
8	Life safety	.557	.404	.348	.367	
14	Opportunity for leisure activities	.416	.382	.334		
Factor2 (F2): Physical (6 items)						
18	Satisfaction with work capacity		.764			
21	Satisfaction with sex life		.743			
19	Self-satisfaction		.692			.448
17	Satisfaction with ability to perform daily living	.365	.630			
15	Ability to get around	.546	.583			
10	Vitality	.339	.507	.389		
Factor3 (F3): Psychological (4 items)						
6	Life meaning			.715		
5	Life enjoyment			.654		
20	Satisfaction of personal relationships			.617		.374
7	Concentration		.423	.533	.304	
Factor4 (F4): Social 1 (2 items)						
26	Negative feeling				.848	
27	The feeling of being respected by others				.352	
Factor5 (F5): Social 2 (1 items)						
22	Satisfaction with friend support					.743
Eigen values		4.640	3.612	2.838	1.718	1.584
Variance explained (%)		20.174	15.706	12.339	7.471	6.887
Total variance explained		62.577				

In the CFA, the fit indices of the models resulting from EFA operations with the WHOQOL-BREF TW were compared with the fit indices of the original 4-model WHOQOL-BREF for TB patients and healthy referents (Table 5). None of the models fit the chi-square fit index, but performed well for the relative chi-square, with values that ranged from 1.73 to 1.94. This was below the recommended cut-off value of 3 [29]. The 2 EFA models had RMSEA values lower than .08, which indicated no significant errors in either model [31]. However, the 2 WHOQOL-BREF models had large RMSEA values, which indicated that these 2 models might contain significant errors. Although some CFI values were less than the required value of 0.9 [30], most were above 0.8. For the AIC values, the EFA models in this study performed better than the WHOQOL-BREF original models. All the fit indices suggested that these EFA models displayed a better fit for both TB patients and healthy referents from Eastern Taiwan.

**Table 5:** Fit indices for the EFA models vs. WHO four-factor models of the original WHOQOL-BREF

**Fit indices**	**Our EFA models**		**WHO 4-factor models**	
	**Healthy referents**	**Patients with TB**	**Healthy referents**	**Patients with TB**
	**4F model**	**6F model**	**4F model**	**4F model**
Number of parameters	71	84	84	84
Discrepancy (*χ*2)	359.648*	370.850*	533.099*	568.568*
(df)	(204)	(215)	(293)	(293)
Relative chi-square	1.763	1.725	1.819	1.941
CFI	0.864	0.862	0.808	0.775
RMSEA	0.077	0.072	0.08	0.082
AIC	501.648	538.85	701.099	736.568

*Notes*: df = degree of freedom; 4F model = 4-factor model; 6F model = 6-factor model; CFI = Comparative fit index; RMSEA = root mean square error; AIC = Akaike index. Ideal fit indices are nonsignificant discrepancy (*p* > .05), relative chi-square < 3, CFI > .9, RMSEA ≦ .08, lower AIC.

* p < .001.

### Convergent validity

Regardless of the sign of a correlation coefficient, Weinberg and Goldberg [37] suggested that Pearson correlation values in the range of .8 to 1.0 are considered strong, in the range of .4 to .6 are considered moderate, and in the range of 0 to .2 are considered weak. Based on this definition, the associations among the WHOQOL-BREF domain scores were moderately correlated and ranged from .56 to .74 for the TB patient group and from .57 to .69 for the healthy referent group (Table 6). The WHOQOL-BREF domain scores were also moderately related to 2 QOL global items--general QOL (G1) and health-related QOL (G2), and ranged from .34 to .69 for the TB patient group (Table 6). For the healthy referent group, the associations among the WHOQOL-BREF domain scores and 2 QOL global items were weak to moderately correlated in the range of .21 to .59 (Table 6). All these correlation coefficients were statistically significant at the .05 significance level.

**Table 6:** Pearson correlations between the WHOQOL-BREF TW domains, and with the two global items

	**Physical**	**Environmental**	**Psychological**	**Social relationship**	**G1**	**G2**
Physical	1	.678**	.688**	.557**	.443**	.530**
Environmental	.606**	1	.713**	.735**	.690**	.339**
Psychological	.688**	.685**	1	.614**	.548**	.576**
Social Relationship	.612**	.574**	.621**	1	.438**	.427**
G1	.408**	.456**	.437**	.357**	1	.337**
G2	.451**	.336**	.592**	.207*	.390**	1

*Notes*: The results of the patient and healthy data are shown in the upper and lower triangle of the correlation matrix, respectively. G1=general quality of life, G2= health related quality of life.

* *p* < .05, two-tailed, ** *p* < .01, two-tailed.

### Discriminant validity

As mentioned, 4 WHOQOL-BREF TW domain scores were highly interrelated (see Table 6), which suggested the use of a multivariate analysis to analyze all these domain scores simultaneously. A series of examinations between the TB patient group and the healthy referent group in the demographic characteristics of participants also revealed significant group differences in both level of education and personal monthly income. These 2 variables were incorporated into the following multivariate analysis.

Using the Wilk Lambda criterion, a MANCOVA test yielded a significant group effect and a significant group*income interaction effect on 4 WHOQOL-BREF TW domain scores, *F* (4, 200) = 3.365, *p* = .011 < .05, and *F* (8, 200) = 2.207, *p* = .026 < .05, respectively (Table 7). The effect of the covariate (level of education) on the WHOQOL-BREF TW domain scores was also significant, *F* (4, 200) = 3.495, *p* = .009 < .05 (Table 7). However, the effect of personal monthly income on these domain scores was not significant, *F* (8, 400) = 1.550, *p* = .138 > .05 (Table 7).

**Table 7:** Multivariate statistics for main effects and interaction effects on the WHOQOL-BREF TW domain scores

**Effect**	**Wilks’ Lambda**	***F***	***df***	***p***	***η***^***2***^
Education**	.935	3.495	4, 200	.009	.065
Group*	.937	3.365	4, 200	.011	.063
Personal income	.941	1.550	8, 400	.138	.030
Group*Personal income*	.917	2.207	8, 400	.026	.042

*p < .05; ** *p* < .01.

Although the results of the univariate analyses indicate that healthy referents had higher scores than TB patients on 3 WHOQOL-BREF domains (physical, environmental, and psychological domains, Table 8). Healthy referents also had higher scores than TB patients in the social relationship domain, but the mean difference between these 2 groups did not reach statistical significance. These analyses revealed the discriminant validity of the WHOQOL-BREF TW on TB patients and healthy referents.

**Table 8:** Means, standard deviations and F values for the WHOQOL-BREF TW domain scores

**Domain**	**TB Patients**		**Healthy referents**		***F***	***η***^***2***^
	**Mean**	**SD**	**Mean**	**SD**		
Physical**	12.7	2.79	14.16	2.17	9.651	0.045
Environmental*	12.71	2.51	13.36	2.55	6.391	0.031
Psychological**	12.41	3.05	13.3	2.24	9.633	0.045
Social Relationship	13.28	2.52	13.74	2.09	1.349	0.007

*p < .05; ** *p *< .01.

## Discussion

The WHOQOL-BREF TW was shown to have acceptable psychometric properties for assessing QOL in healthy Taiwanese people. In this study, the internal consistency of the healthy participants was good. The EFA of the data from healthy referents generated a factor structure similar to the WHO’s original four-factor model. This finding confirmed the construct validity of the WHOQOL-BREF TW for assessing the general Taiwanese population. However, when assessing Taiwanese TB patients, the resulting EFA factor structure was rather different from the WHO’s original factor structure. This finding brings our attention to the appropriateness of assessing the WHOQOL-BREF TW in Taiwanese TB patients.

In the present study, the construct validity of the WHOQOL-BREF TW among Taiwanese TB patients was not fully supported. For instance, the top two factors of the WHOQOL-BREF TW among Taiwanese TB patients were self-confirmation and social support. The self-confirmation factor seemed to suggest that having a sense of self-assurance was a crucial reason for Taiwanese TB patients to maintain their quality of life after having this disease. TB patients with high self-confirmation may feel satisfied with the self and less likely considered themselves inferior to other people after having TB.

In Taiwan, TB is a regulated disease. Patients with TB may be hospitalized and isolated from the general public until they are not infectious. Under this circumstance, TB patients may not only suffer from disease-related health problems, but also from psychological dissatisfaction and social stigma [6]. They are likely to feel dissatisfied about their life quality after having TB. Therefore, social support may be an important factor for Taiwanese TB patients to maintain their QOL.

Recently, the idea of social stigma related to patients with TB has been studied [6,8,38]. Two different types of stigma associated with TB are public discrimination and the internalized stigma that patients feel after contracting TB [6]. To maintain QOL, TB patients need to develop mechanisms to protect themselves from these two stigmas. In this study, the top two EFA factors (i.e., self-confirmation and social support) of the WHOQOL-BREF TW among Taiwanese TB patients seem to support this argument. Self-confirmation and social support may become two important mechanisms that protect TB patients from TB-related stigmas.

In the present study, although the EFA factor structure of the WHOQOL-BREF TW among Taiwanese TB patients yielded a different factor structure from the WHO’s original one, this finding indicated essential QOL components among TB patients that differed from the healthy referent group. Future research needs to recruit another TB patient sample to validate the factor structure of the WHOQOL-BREF among Taiwanese TB patients as well as TB patients in different countries.

With regards to the reliability of the WHOQOL-BREF TW, good internal consistency reliability was found in both TB patients as well as healthy referents. Due to limited fund and resources, in this study, only TB patients were administrated three times to evaluate the effect of the TB patient treatment plan. Test-retest reliability among TB patients was not reported because it could be affected by the effect following TB treatment, which became the limitation of this study.

Finally, the relative small sample size would be another limitation of this study. Due to limited TB patient population in Eastern Taiwan, TB patients recruited in this study were only 140 people that were below the general expected sample size for conducting factor analysis. A longitudinal study or a cross-sectional study to recruit at least 300 to 600 TB patients would be our next step to reexamine the validity issue (especially construct validity) for assessing the WHOQOL-BREF TW in Taiwanese TB patients.

## Conclusion

This study found good internal consistency reliability of the WHOQOL-BREF TW in the healthy referent group, and the factor structure generated from these healthy referents was similar to the WHO’s original model. This finding indicates the appropriateness of applying this instrument in Taiwanese healthy people. Although good internal consistency reliability of the WHOQOL-BREF TW was found in the TB patient group, the factor structure generated from this group differed from the WHO’s original four-domain structure. This finding highlighted the validity concern when applying this instrument to assess patients with TB.

## Competing interests

The authors declare that they have no competing interests.

## Authors’ contributions

WSC contributed to conception and design, acquisition of data, analysis and interpretation of data, and involved in writing the manuscript and responding the reviewers’ comments. YLL contributed to analysis and interpretation of data and writing the manuscript and responding the reviewers’ comments. MCY devoted himself to providing the critical and important intellectual content and revising the manuscript for publication. All authors read and approved the final manuscript.

## Pre-publication history

The pre-publication history for this paper can be accessed here:

http://www.biomedcentral.com/1471-2458/12/630/prepub
